# The NAC transcription factor family in maritime pine (*Pinus Pinaster*): molecular regulation of two genes involved in stress responses

**DOI:** 10.1186/s12870-015-0640-0

**Published:** 2015-10-24

**Authors:** Ma Belén Pascual, Francisco M. Cánovas, Concepción Ávila

**Affiliations:** Departamento de Biología Molecular y Bioquímica, Facultad de Ciencias, Campus Universitario de Teatinos, Universidad de Málaga, 29071 Málaga, Spain

**Keywords:** *Pinus pinaster*, *NAC* gene family, Stress, Methyl jasmonate, Promoter

## Abstract

**Background:**

NAC transcription factors comprise a large plant-specific gene family involved in the regulation of diverse biological processes. Despite the growing number of studies on NAC transcription factors in various species, little information is available about this family in conifers. The goal of this study was to identify the NAC transcription family in maritime pine (*Pinus pinaster*), to characterize ATAF-like genes in response to various stresses and to study their molecular regulation.

**Methods:**

We have isolated two maritime pine *NAC* genes and using a transient expression assay in *N. benthamiana* leaves estudied the promoter jasmonate response.

**Results:**

In this study, we identified 37 *NAC* genes from maritime pine and classified them into six main subfamilies. The largest group includes 12 sequences corresponding to stress-related genes. Two of these *NAC* genes, *PpNAC2* and *PpNAC3,* were isolated and their expression profiles were examined at various developmental stages and in response to various types of stress. The expression of both genes was strongly induced by methyl jasmonate (MeJA), mechanical wounding, and high salinity. The promoter regions of these genes were shown to contain *cis*-elements involved in the stress response and plant hormonal regulation, including E-boxes, which are commonly found in the promoters of genes that respond to jasmonate, and binding sites for bHLH proteins. Using a transient expression assay in *N. benthamiana* leaves, we found that the promoter of *PpNAC3* was rapidly induced upon MeJA treatment, while this response disappeared in plants in which the transcription factor NbbHLH2 was silenced.

**Conclusion:**

Our results suggest that *PpNAC2* and *PpNAC3* encode stress-responsive NAC transcription factors involved in the jasmonate response in pine. Furthermore, these data also suggest that the jasmonate signaling pathway is conserved between angiosperms and gymnosperms. These findings may be useful for engineering stress tolerance in pine via biotechnological approaches.

**Electronic supplementary material:**

The online version of this article (doi:10.1186/s12870-015-0640-0) contains supplementary material, which is available to authorized users.

## Background

Conifers are the most important group of gymnosperms, dominating large ecosystems in the Northern Hemisphere, and they are also of great economic importance, as they are intensively used for timber, fuelwood, resins and paper production [1]. During millions of years of co-existence with changing environmental conditions, competing plants, potential pests and foraging animals, conifers have evolved potent and effective defense mechanisms. These mechanisms include structural, morphological or physical barriers, such as resin canals, calcium oxalate structures, sclereid cells and lignin, and/or chemical defences, which include the production of phenolics or volatile and non-volatile terpenoid compounds [2]. In previous decades, it was discovered that several plant phytohormones such as jasmonic acid (JA), ethylene (ET) and salicylic acid (SA) are involved in complex signalling cascades and in the synthesis of chemical defenses [3]. In particular, the Me-JA pathway has been found to be closely related to the wounding response to defoliating caterpillars, budworms and bark beetles [4–6]. In contrast to our detailed knowledge of the structures and chemical response related to stress, there is little information on the molecular mechanisms that enable plants to cope with environmental changes.

Maritime pine (*Pinus pinaster Aiton*) is particularly tolerant to abiotic stresses, displaying relatively high levels of intraspecific variability [7]. Maritime pine is also used as a model tree for conifer genomic research in Europe [8, 9] and the emergence of next-generation sequencing (NGS) has facilitated the *de novo* assembly of the transcriptome [10]. Sequencing data are available at SustainpineDB (http://www.scbi.uma.es/sustainpinedb/sessions/new).

A total of 877 transcription factors (TF) distributed into 30 families on the basis of conserved structural domains involved in DNA binding were identified in the maritime pine transcriptome [10]. The number of TF in maritime pine is similar to that previously reported for white spruce [11] but smaller than that reported for angiosperm species [10].

Regulation of gene expression plays a fundamental role in plant response to environmental stimuli. Recently accumulated evidence demonstrates that numerous families of TF, including the DREB, bZIP, MYB, zinc-finger, WRKY and NAC families, directly or indirectly regulate plant defenses and stress responses [12–18].

The NAC family is one of the largest plant-specific transcription factor families and is represented by 105 genes in *Arabidopsis*, 140 in rice, 110 in potato, 163 in poplar and 32 in white spruce. NAC proteins have a highly conserved N-terminal DNA-binding domain comprising nearly 160 amino acid residues divided into five subdomains (A-E). The function of the NAC domain has been associated with nuclear localization, DNA binding and the formation of homo or heterodimers with other NAC domain-containing proteins [19]. In contrast, the C-terminal region is highly divergent and contains a transcriptional regulatory domain [20]. The NAC factors regulate the expression of genes involved in processes such as shoot apical meristem development [21–23], floral morphogenesis [22, 24], lateral root development [25], leaf senescence [26, 27], regulation of cell cycle [28, 29], hormone signaling [25, 28, 30, 31], grain nutrient remobilization [32], xylogenesis, fiber development and wood formation [33–35]. NAC proteins also participate in plant responses to abiotic and biotic stresses [36, 37].

Several NAC proteins have been characterized in *Arabidopsis*, rice, soybean and cotton and have the potential to improve biotic and abiotic stress tolerance in plants. The overexpression of ANAC019, ANAC055 and RD26 (ANAC072) in *Arabidopsis* upregulated the expression of stress-inducible genes and improved the drought and salt tolerance of plants [38]. ATAF1 and ATAF2 in *Arabidopsis*, and HvNAC6 in barley play important roles in response to drought and pathogen stresses [31, 39–41]. ATAF1 acts as a negative regulator of ABA signaling but induces MeJA/ET-associated defense signaling marker genes [31]. Conversely, ATAF2 expression is induced by dehydration, MeJA and SA, independently of ABA [40]. In rice, the overexpression of OsNAC1 and OsNAC5 enhances drought and salt tolerance and grain yield under field conditions [42, 43].

The structural characterization of members of the family of NAC transcription factors in angiosperms has greatly increased in past few years; however, the functions of most of most of these TF remain unknown. Limited information is available regarding NAC proteins in gymnosperms [23, 44]. In this study, we identified a total of 37 NAC domain-containing TF in *P. pinaster*. Detailed analyses, including those of sequence phylogeny, conserved motifs and promoter analysis were performed. Furthermore, we have analyzed the expression patterns of two *P. pinaster* NAC genes, *PpNAC2* and *PpNAC3*, which clustered with *Arabidopsis ATAF1* and *ATAF2* genes. We have identified its responses to treatments with high salinity, low temperature, wounding, MeJA and ABA. Both genes were rapidly and strongly induced upon MeJA treatment and/or wounding.

Furthermore, we performed *in silico* and *in vivo* analyses of the PpNAC3 regulatory region. In a transient expression approach using *Nicotiana benthamiana* leaves, the expression of *PpNAC3* was regulated by bHLH MYC jasmonate-responsive transcription factors. This suggests a conserved mechanism in two phylogenetically distant species.

## Results

### Identification and phylogenetic analysis of members of the NAC family

The conserved DNA-binding domain of known NAC proteins was used as a query to identify the *NAC* genes in the maritime pine database (SustainpineDB). A total of 37 putative *NAC* genes were identified. We have annotated all the NAC domain-encoding genes as PpNACxx, where Pp is the species initials (*Pinus pinaster*) and xx is the number given in the ordered identification in the SustainpineDB.

The identified NAC genes in *P. pinaster* encode proteins with an average of 409 amino acids. Detailed information about the pine NAC genes identified in the present study, including accession numbers, similarities to the *Arabidopsis* putative orthologues, and the protein sequences, is provided in Additional file 1.

**Fig. 1:**
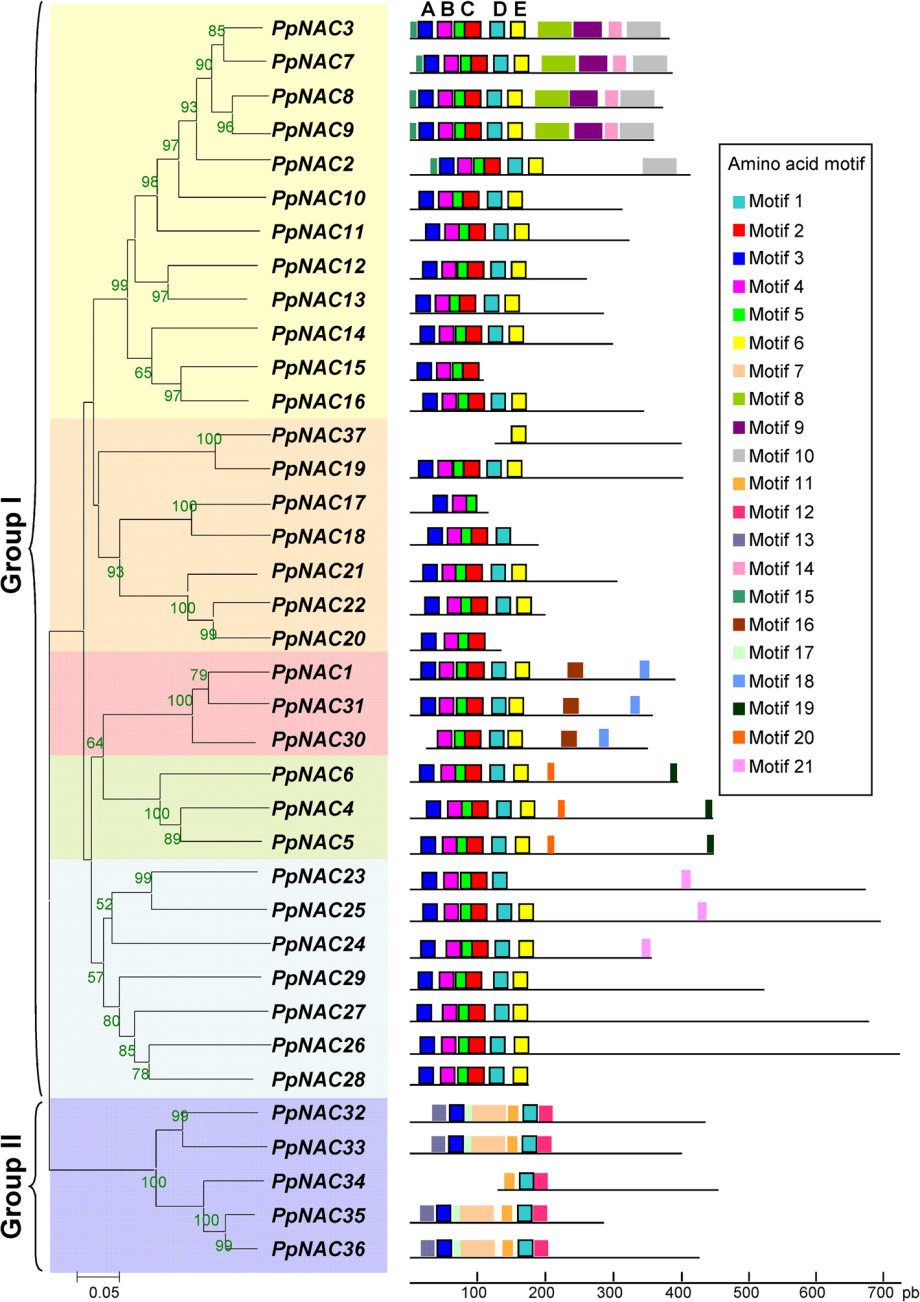
The NAC protein family in *P. pinaster.* Multiple alignments of the 37 proteins encoded by *NAC* genes from *P. pinaster* were executed by Clustal X, and the phylogenetic tree was constructed using MEGA 4.0 via the Neighbor-Joining (NJ) method with 1000 bootstrap replicates. Percentage bootstrap score higher than 50 % and subfamily classification are indicated. Amino acid motifs in the NAC proteins (1 to 21) are represented by colored boxes (Additional file 2). The black lines indicate relative protein lengths

The program Clustal X version 1.83 was used for multiple sequence alignments of the protein sequences of *P. pinaster.* The results indicated that the *P. pinaster* NAC family can be classified into two groups, based on similarities in the structure of the DNA-binding domain: Group I, which could be subdivided into five clusters, and Group II, composed of a single cluster of five NAC proteins (Fig. 1).

To further study the diversification of the NAC family in pine, we predicted conserved putative motifs using the MEME program [45]. Twenty-one protein motifs containing 6 to 50 residues were identified. To simplify, we considered only those motifs that are present in more than half of the members of a cluster. Most of the closely related members in the phylogenetic tree had common motif compositions (Fig. 1). Five subdomains, A-E, were previously defined in the N-terminal region of NAC proteins [20, 46]. We assigned Motif 3 to subdomain A, Motif 4 to subdomain B, Motif 5 and Motif 2 to subdomain C, Motif 1 to subdomain D and Motif 6 to subdomain E (Fig. 1 and Additional file 2). The subdomain distribution on the N-terminal region of on different NAC proteins is showed in the Additional file 3. Most of the maritime pine NAC proteins contain subdomains A to E in the DNA-binding domain; however members of Group II only contain subdomains A and D and lack subdomains B, C and E. These proteins contain the Motif 7 in the N-terminal region, which seems to have replaced Motifs 2, 4 and 5 from most *PpNAC* sequences. Group II proteins also contain Motifs 11, 12 and 13 in the NAC domain. The C-terminal region is highly divergent, although we were able to identify certain specific motifs present in NAC proteins from specific clusters of Group I (Additional file 2). Motifs 8, 9, 10 and 14 were present in the NAC proteins included in the largest cluster. The biological significance of most of the putative motifs is unknown and requires further investigation, but it is tempting to speculate that the structural homology may be related to function.

**Fig. 2:**
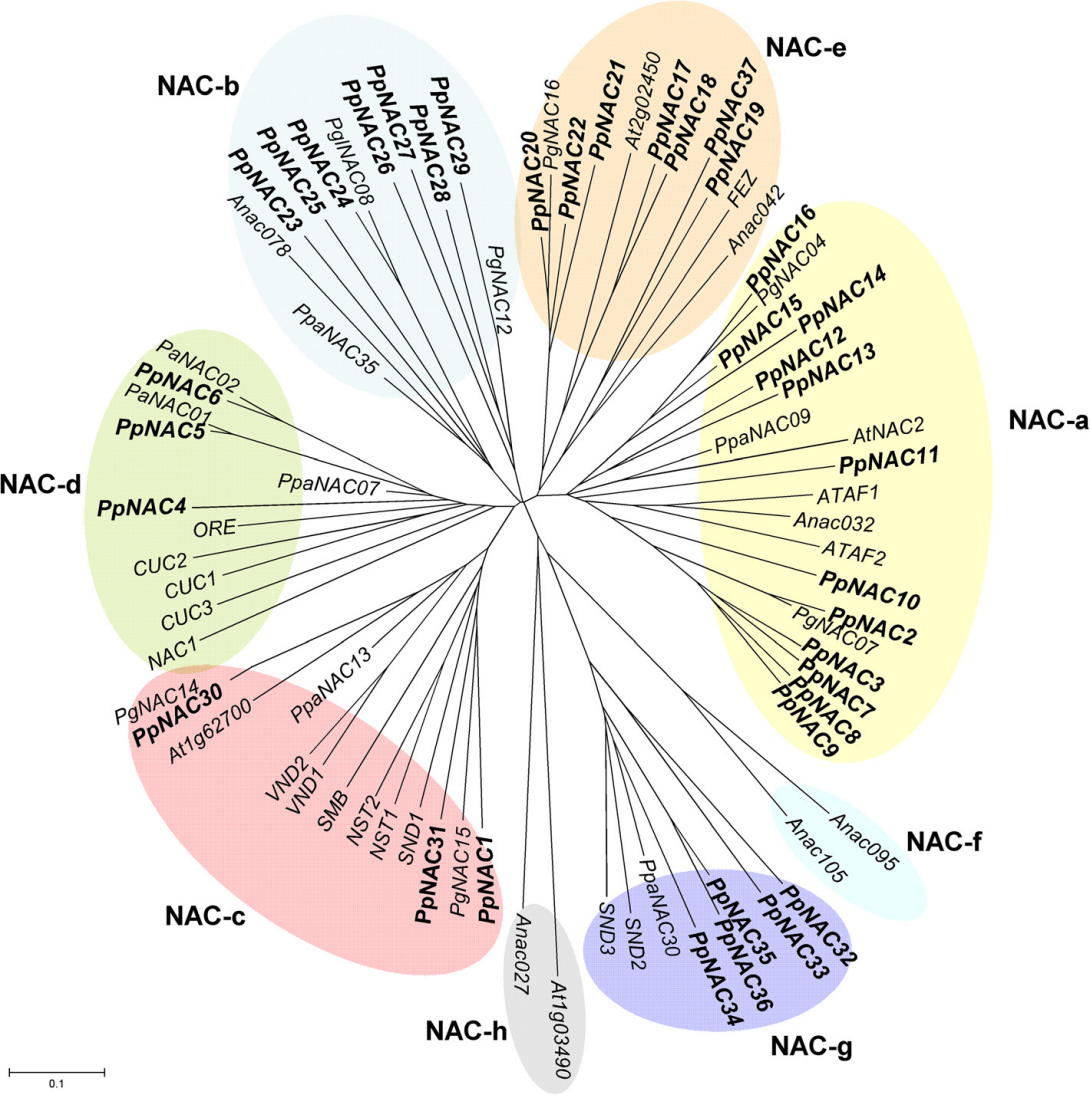
Phylogenetic analysis of *Arabidopsis thaliana*, *Physcomitrella patens*, *Picea abies*, *Picea glauca* and *Pinus pinaster* NAC proteins. The phylogenetic tree was constructed with full-length NAC amino acid sequences using the Neighbor-Joining method. Major clades previously identified by [46] are indicated (*a*-*h*). The accession numbers of sequences used in the analysis are available in Additional file 4: Table S2

To explore the phylogenetic relationships of maritime pine NAC factors and other members of this family in plants the sequences obtained in this work and the full-length protein sequences of *P. glauca, P. abies, A. thaliana* and *Physcomitrella patens* were compared. Figure 2 shows that the pine NAC family can be classified into six subfamilies (a-h) according to [46]. Group I comprises the NAC-a, NAC-b, NAC-c, NAC-d and NAC-e subfamilies, whereas Group II comprises subfamily NAC-g. The clade NAC-a is the largest in *P. pinaster* including 12 sequences. This clade includes genes that are phylogenetically close to stress-related genes such as ATAF1 and ATAF2 [41] and PpaNAC09, PgNAC04 and PgNAC07 (Additional file 4) [23, 46].

The clade NAC-b includes seven *P. pinaster* sequences with similarity to proteins with transmembrane motifs in their C-terminals that mediate either cytokinin signalling during cell division or endoplasmatic reticulum stress responses [28, 47]. The NAC-e clade includes seven *P. pinaster* sequences and the *FEZ* gene, which has been demonstrated to be associated with the orientation of cell division in root stem cells [29]. Three *P. pinaster* sequences were grouped in the NAC-c clade with the NAC involved in vascular development, such as VND1/2, NST1/2, SND1 and SMB [48–50]. Three *P. pinaster* sequences are clustered in clade NAC-d together with CUC1/2/3 and ORE genes from *Arabidopsis* [51–54], and PaNAC1 and PaNAC2 genes from *P. abies* [23]; the genes of this clade may be involved in organ initiation and differentiation. Five sequences clustered with clade NAC-g together with the SND2 and SND3 genes of *Arabidopsis*; these genes are involved in the secondary cell wall transcriptional network [55]. Clades NAC-f and NAC-h did not harbor any *P. pinaster NAC* genes. The functions of most NAC genes that clustered with these two clades still remain unknown, but it is significant that there is no representative of these sub-families in maritime pine.

### Cloning and molecular characterization of two NAC genes in *P. pinaster*

Based on previous microarray data obtained in our laboratory, two maritime pine *NAC* genes in the NAC-a subfamily, *PpNAC2* and *PpNAC3* (Fig. 2), were selectedfor functional characterization. *PpNAC2* and *PpNAC3* cDNA were cloned using PCR and fully sequenced. These genes displayed 75 % sequence identity to *Arabidopsis* ATAF1 and ATAF2, which are reported to be NAC transcription factors with biological functions in abiotic and biotic stress responses [40, 41]. The cDNA for *PpNAC2* was 1170 bp in length and contained an ORF encoding a protein of 387 amino acids whereas the *PpNAC3* cDNA was 1152 bp in length with a deduced amino acid sequence of 383 amino acids. Regulatory sequences upstream of the initiation codon of *PpNAC2* (of 661 bp) and *PpNAC3* (1115 bp) were isolated using a Genome Walking approach.

### *PpNAC2* and *PpNAC3* spatial and temporal organ-specific expression

**Fig. 3:**
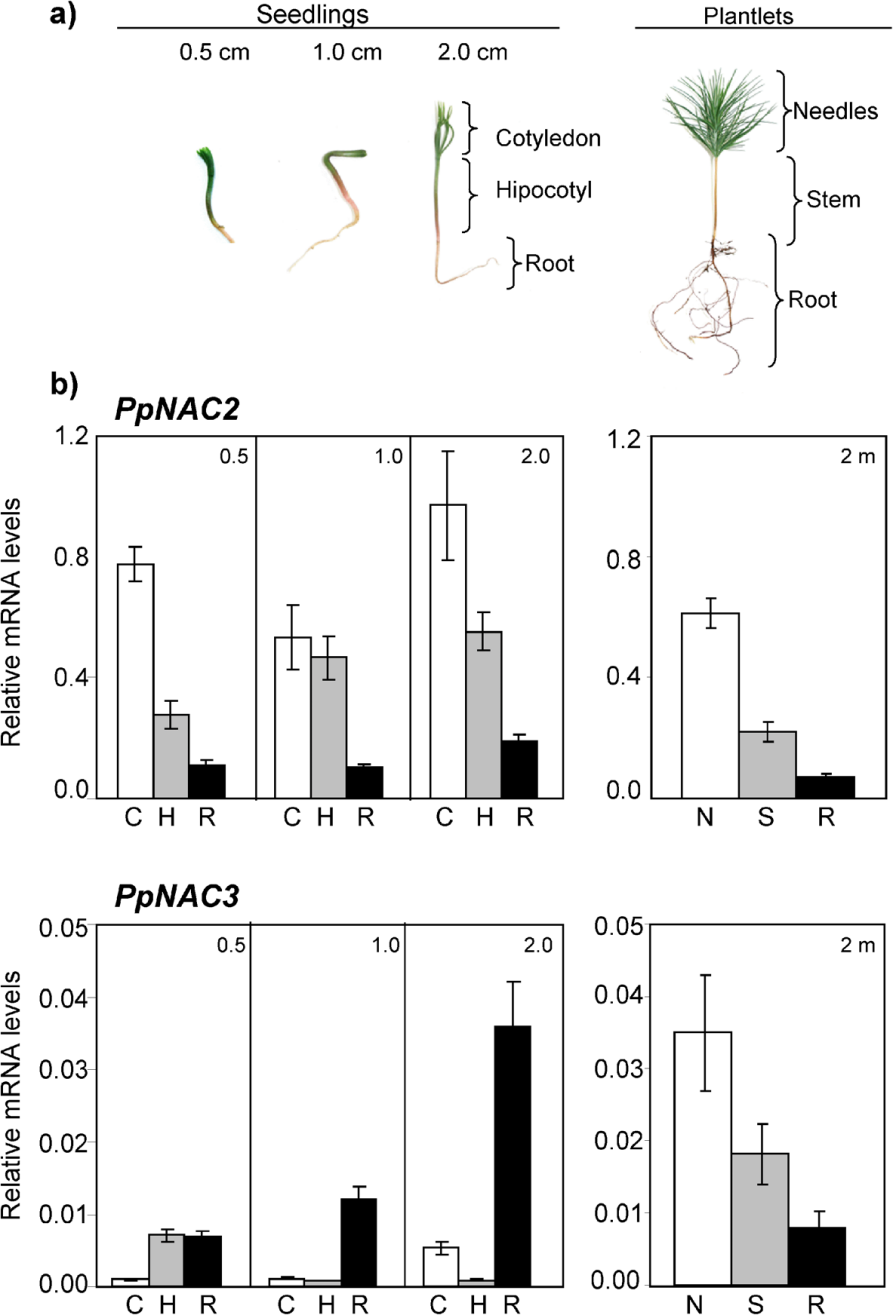
Expression patterns of *PpNAC2* and *PpNAC3.* **a** Representative images of the pine seedlings with cotyledons 0.5, 1.0 and 2.0 cm in length and 2-months-old plantlets used in this study. **b** qPCR analysis of *PpNAC2* and *PpNAC3* transcripts in different organs of the *P. pinaster* seedlings and plantlets. Total RNA was extracted from different samples and reverse transcribed. The cDNA was amplified using specific primers for each gene, as described in Additional file 4: Table S1. The expression data were normalized using a geometric mean of the reference genes (*ACT*, *40S* and *EF1α*). Analysis was performed three times on three independent biological samples, and the mean values ± SE are indicated. (C) Cotyledons, (N) Needles, (H) Hypocotyl, (S) Stem and (R) Root

To determine the spatial and developmental expression patterns of these genes in *P. pinaster,* total RNA was extracted from various plant organs, and their relative abundance was analyzed by quantitative PCR (qPCR). Gene expression of *PpNAC2* and *PpNAC3* was analyzed in seedlings bearing cotyledons 0.5, 1.0 and 2.0 cm in length and in 2-month-old plantlets (Fig. 3a). In the seedlings, *PpNAC2* transcripts were particularly abundant in the cotyledons and hypocotyls, while much lower levels were found in roots. In contrast, *PpNAC3* was predominantly expressed in the roots of the seedlings during development. Interestingly, *PpNAC2* and *PpNAC3* exhibited a similar tissue-specific pattern of expression in 2-month-old plantlets with maximum levels of transcripts detected in the needles (Fig. 3b). Notably, the expression level of *PpNAC2* was an order of magnitude higher than of *PpNAC3*. The transcript levels were normalized to the expression levels of reference genes, as described in the Methods section.

### *PpNAC2* and *PpNAC3* expression in response to abiotic stresses and hormone treatments

**Fig. 4:**
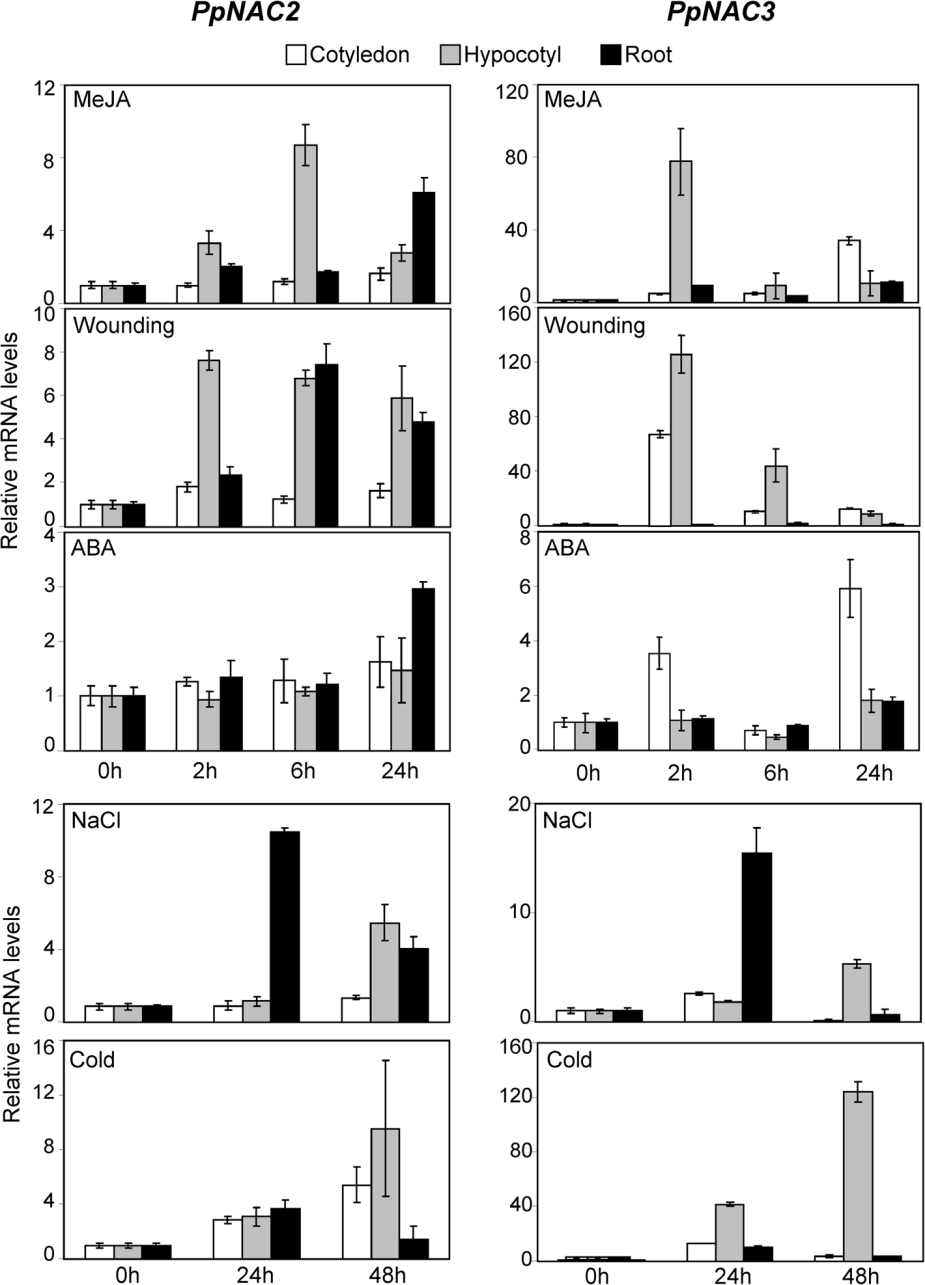
Stress-responsive transcript profiles of *PpNAC2* and *PpNAC3* as determined by qPCR. Transcript accumulation in cotyledons, hypocotyls and roots from 3-week-old seedlings in response to 100 μM MeJA, mechanical wounding and 50 μM ABA (after 0, 2, 8 and 24 h); in response to 250 mM NaCl (after 0, 24 and 48 h) and exposure to cold (after 0, 24 and 48 h). Data were normalized using a geometric mean of the reference genes (*ACT*, *40S* and *EF1a*). Mean values ± SE are shown for three independent experiments

To test whether *PpNAC2* and *PpNAC3* are stress-responsive genes, we performed a qPCR analysis on total RNA isolated from the cotyledons, hypocotyls and roots of seedlings subjected to different stresses. The expression *PpNAC2* and *PpNAC3* was upregulated in response to MeJA and wounding (Fig. 4, MeJA, Wounding). However, *PpNAC2* exhibited a sustained response in hypocotyls and roots during a period of 24 h whereas a short-term response was observed for *PpNAC3* at 2 h, preferentially in hypocotyls. The response to ABA was only detected 24 h after treatment, and it was observed exclusively in roots for *PpNAC2* and in cotyledons for *PpNAC3* (Fig. 4, ABA). In contrast, *PpNAC2* and *PpNAC3* responded similarly to NaCl and cold treatments (Fig. 4, NaCl, Cold). It is worth mentioning that the magnitude of the response to the different treatments was always higher for *PpNAC3* than for *PpNAC2.* Specifically, the observed induction of *PpNAC3* was aproximately 10-fold that of *PpNAC2* in response to MeJA, wounding and cold.

### *PpNAC2* and *PpNAC3* promoters contain *cis*-elements involved in biotic and abiotic stress

**Fig. 5:**
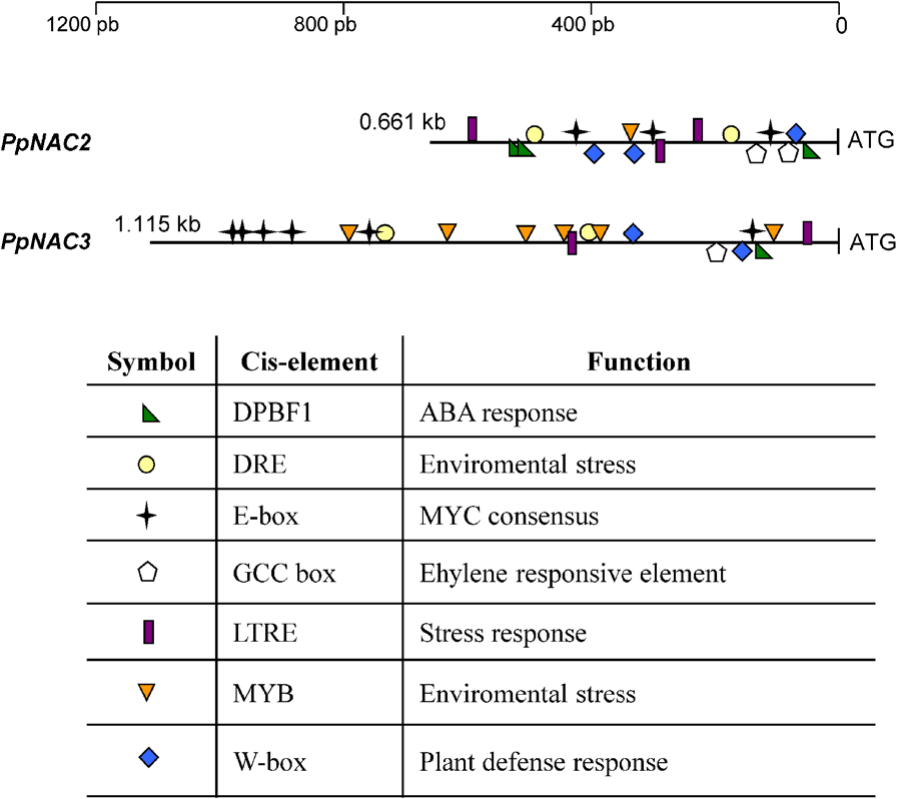
Distribution scheme of putative *cis*-acting elements in the *PpNAC2* and *PpNAC3* gene promoters. The 5′regulatory region of these genes was analyzed for the presence of putative *cis*- acting regulatory elements using the plant *cis*-acting regulatory DNA elements (PLACE) database (http://www.dna.affrc.go.jp/PLACE/), and some stress-responsive and hormonal regulation *cis*-elements listed in the PLACE database were mapped. The relative positions are presented with respect to the first base of the translation start site (ATG)

To further explore the regulation of these *NAC* genes in maritime pine the 5′-upstream sequences of *PpNAC2* and *PpNAC3* were subjected to a search in the PLACE database (https://sogo.dna.affrc.go.jp/cgi-bin/sogo.cgi) [56] to identify putative *cis*-regulatory elements. The analysis showed that both promoters had similar stress responsive *cis*-elements such as DPBF1 (ABA-responsive element), W-boxes, GCC-boxes, MYB binding sites and W-boxes (Fig. 5). This analysis also revealed that 3 E-boxes (CANNTG) are located in the 661 nt of *PpNAC2* promoter sequence, and 6 E-boxes are located in the 1,115 nt of *PpNAC3* promoter sequence. E-boxes have been found in the promoters of defense genes in plants [57]. Furthermore, E-boxes are well-characterized binding sites for bHLH TFs in plants and are considered the cognate element for AtMYC2 binding, which has an important role in the activation of early jasmonate-responsive genes in *Arabidopsis* [58, 59]. These elements are commonly found in the promoters of genes that respond to MeJA [59, 60].

### Functional analysis of the *PpNAC3* promoter in *N. benthamiana*

Because the production of stably expressing conifer lines takes about one year and the selection of transgenic lines is a laborious process, we performed transient expression assays in *Nicotiana* as a simple and efficient method for the quantitative analysis of plant promoters *in vivo* [61, 62]. The promoter of *PpNAC3* was selected for functional analysis based on its observed response to stress (Fig. 4).

**Fig. 6:**
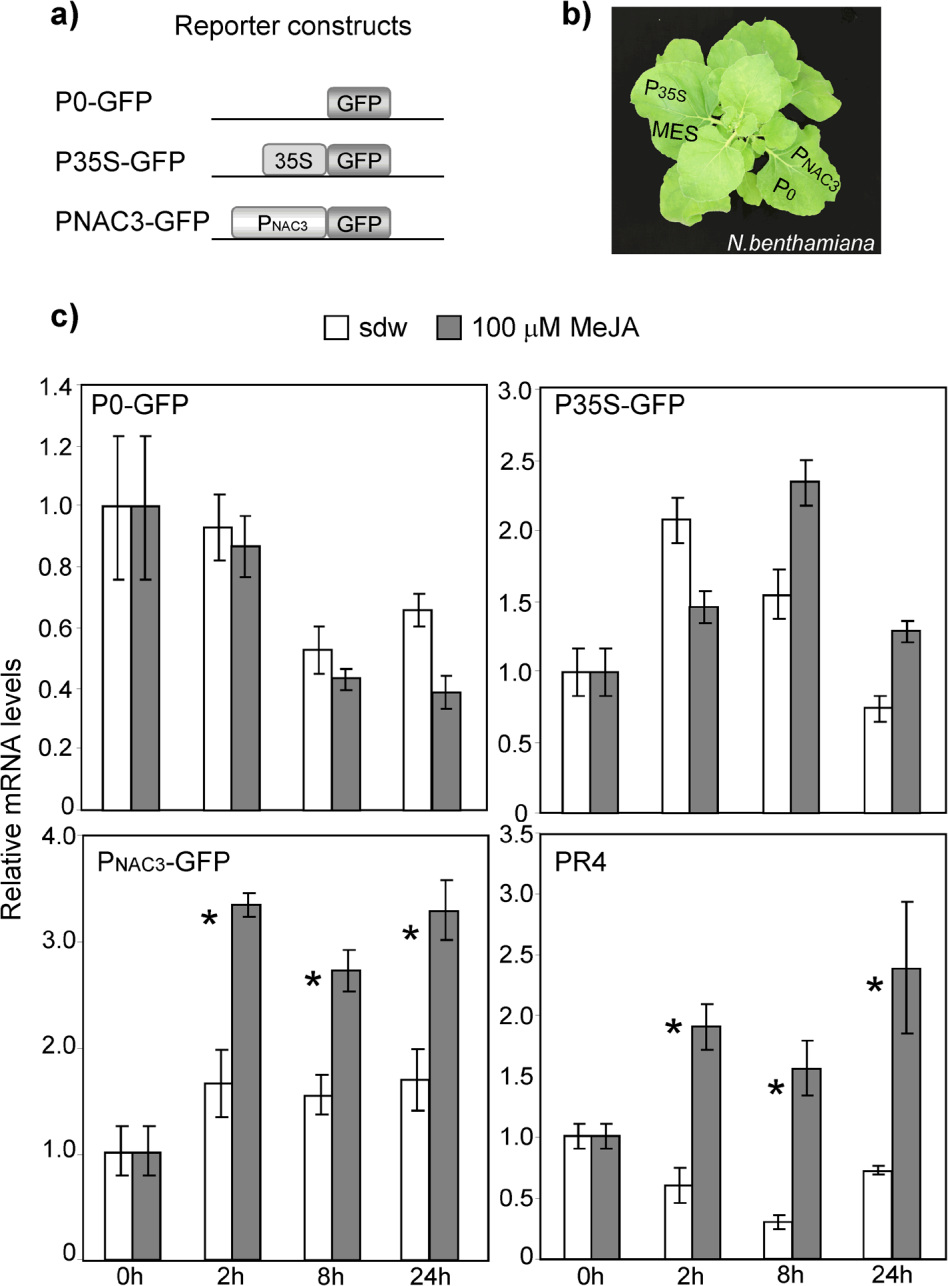
Transient expression of the *PpNAC3* promoter fused to GFP in *N. benthamiana* leaves. **a** Diagram of constructs used for the *Agrobacterium*-mediated transient expression assay in *N. benthamiana* leaves. **b** Plants of *N. benthamiana* used for agroinfiltration. The constructs were agroinfiltrated as shown in the image. We used different leaves of the same plant for each construct, and the experiment was performed in triplicate. After 48 h, the leaves were treated with 100 μM MeJA or with water as a control. **c** Total RNA was isolated from *N. benthamiana* leaves and *GFP* or *PR4* expression was studied at 2, 8 and 24 h after MeJA or water treatment using qPCR. Data were normalized to *NbActin* as a reference gene. Expression levels are relative to the values at time zero. The mean values ± SE are indicated. Asterisks indicate significant differences between the two treatments

A construct containing the promoter region of *PpNAC3* (1115 bp) was fused to the GFP reporter gene in the binary vector p35S-GFP, replacing the 35S promoter and generating P_NAC3_-GFP. p35S-GFP was used as the positive control, and MES buffer and p35S-GFP without the 35S promoter (p0-GFP) were used as the negative controls (Fig. 6a). *N. benthamiana* leaves were agroinfiltrated with *Agrobacterium* containing the various constructs (Fig. 6b). MeJA activated the transcription of the reported gene driven by the *PpNAC3* promoter (Fig. 6c, P_NAC3_-GFP). In comparison with water-treated leaves, an approximately 3-fold increase in GFP expression was observed after 2, 8 and 24 h of the MeJA treatment. In contrast, leaves infiltrated with the negative control or with MES buffer showed no increase in GFP expression (Fig. 6c, P0-GFP). The leaves infiltrated with the positive control exhibited a transient increase in GFP expression regardless of whether they were treated with MeJA or water (Fig. 6c, P35S-GFP). As an additional control, the expression level of the endogenous *PR4* gene was analyzed as a marker for the jasmonic acid-dependent signalling pathway. As shown in Fig. 6c (PR4), the expression of PR4 was rapidly induced by MeJA, and the observed profile was quite similar to that mediated by the *PpNAC3* promoter.

### Regulation of expression mediated by the *PpNAC3* promoter in *NbbHLH2* silenced plants

To further investigate the jasmonate-mediated regulation of the *PpNAC3* promoter in *N. benthamiana* we used a VIGS approach to silence bHLH MYC proteins. Previous reports have shown that promoter sequences that are recognized and bound by MYC2 proteins are highly conserved between different groups of plants [58, 63]. In *N. benthamiana*, the proteins *NbbHLH1* and *NbbHLH2* can bind an E-box element in the *PMT* promoter [64] and activate it [65].

**Fig. 7:**
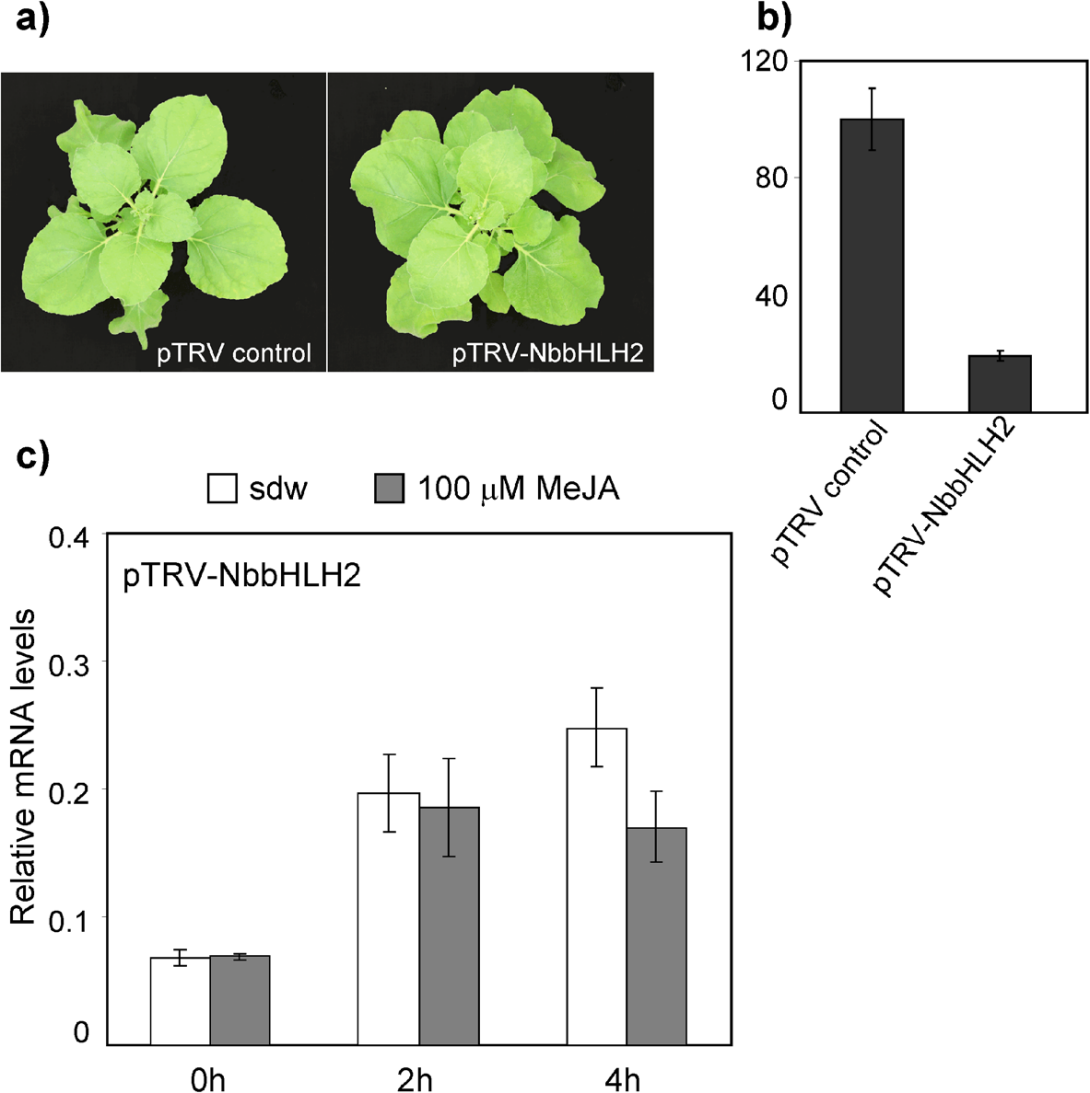
Functional analysis of the *PpNAC3* promoter in VIGS-silenced *NbbHLH2* plants. **a** Photographs of 6-week-old control (pTRV-control) and *NbbHLH2-*silenced plants. **b** The relative expression level of *NbbHLH2* was determined by qPCR in pTRV control and *N. benthamiana* plants with *NbbHLH2* suppression. **c** GFP expression driven by the *PpNAC3* promoter in pTRV-*NbbHLH2-*silenced plants. After 48 h of agroinfiltration with the P_NAC3_-GFP construct, the leaves were treated with either 100 μM MeJA or with water as a control. *NbActin* was used as the endogenous qPCR control. The error bars represent the SD from three biological replicates measured in triplicate

Using RT-PCR and specific primers, we obtained a 400 bp fragment corresponding to the 5′region of the open reading frame of the *NbbHLH2* gene. The fragment was inserted into the vector pTRVGW (pTRV-NbbHLH2), a Gateway-compatible tobacco rattle virus vector [66]. Four weeks later, NbbHLH2-silenced plants showed no change in morphology compared to pTRV control plants (Fig. 7a). Silencing of the endogenous phytoene desaturase gene, *NbPDS*, was used as a control for the effectiveness of VIGS. The degree of silencing was assessed by qPCR, demonstrating that *NbbHLH2* transcript abundance was reduced by approximately 85 % compared to that of pTRV control plants (Fig. 7b).

The MeJA regulation of the *PpNAC3* promoter was studied in *N. benthamiana* leaves after silencing *NbbHLH2.* After 48 h of agroinfiltration with the P_NAC3_-GFP construct, the leaves were treated with MeJA, and GFP expression was determined at 0, 2 and 4 h. As shown in Fig. 7c, in *N. benthamiana* leaves with silencing of the endogenous *NbbHLH2* gene, the level of the GFP transcripts were not altered after MeJA treatment.

## Discussion

Forest trees are routinely exposed to environmental stresses. Current and predicted climatic conditions, such as prolonged drought, increased salinization of soil and water, and high-temperature episodes, are a serious threat to forest productivity worldwide, affecting tree growth and survival. An understanding of how forest trees adapt to hostile environmental conditions is necessary to sustain productivity and to meet future demand for forest-derived products. Current efforts to use molecular analyses and genetic engineering to improve abiotic stress tolerance depend on a thorough understanding of plant signaling pathways involved in the response to stress, as well as on the identification of key regulatory proteins [67].

In this work, we have identified 37 non-redundant NAC domain proteins in the *P. pinaster* genome. This number is close to those previously reported for *Picea glauca* (36) [11, 44] and *Physcomitrella patens ssp. patens* (35) [68] but is substantially smaller than the reported number in angiosperm species: 117 in *Arabidopsis,* 151 in rice, 163 in poplar, 189 in eucalyptus and 152 each in soybean and tobacco [69–74]. Similar findings have also been reported for members of other TF gene families in conifers, such as the Dof gene family, which contains ten members in maritime and loblolly pines [75], noticeably fewer than the numbers of Dof genes in angiosperms [76, 77]. These data suggest that the NAC gene family, as has occurred in other TF families, has expanded and diversified in angiosperms by gene duplication, creating paralogous genes with a high degree of sequence similarity and functional redundancy.

The *P. pinaster* NAC proteins can be phylogenetically clustered into two subgroups based on the similarities of their DNA-binding domains. The Group I has 31 members and can be further classified into five subgroups, and Group II consists of six NAC proteins (Fig. 1). The conserved motif identified using the MEME program defines six subfamilies of *P. pinaster* NAC proteins, which was consistent with our phylogenetic analysis. Furthermore, of the five subdomains (A-E) identified in the N-terminal region of all proteins in Group I, we identified four conserved motifs (Motifs 7, 11, 12 and 13), which were also located in the N-terminal regions of Group II NAC proteins. Specifically Motif 7 is similar to Motif 9, which has been found in a minority of NAC proteins of various plants [74]. This motif is homologous to the NAM domain (PF02365) and appears to replace Motifs 2, 4 and 5 (Fig. 1*b*). Most of the conserved motifs located in the C-terminal regions are novel, but some of them have been found to be related despite performing different functions. For example, Motif 10 has been previously described as a transcriptional activation motif [20, 78], and it is present in stress-related genes. Motifs 19 and 20, conserved among the members of one subfamily, correspond to the W-motif and L-motif, respectively, as previously described for the C-terminal domain of the CUC subfamily [23, 52, 79]. However, the biological significance of most of the protein motifs is currently unknown and therefore remains to be further investigated.

Shen et al. (2009) carried out a genome-wide bioinformatics survey on plant NAC domain TFs from different plant species, without including any gymnosperms in the analysis. The phylogenetic analyses of *P. pinaster* NAC proteins together with the characterized NAC proteins from other plants suggest that all NAC sequences from *P. pinaster* could be included in the different clades (Fig. 2*a*), as previously indicated by [46]. However, clades f and h did not harbor any *P. pinaster* NAC proteins. Interestingly, the NAC-f subfamily was also absent in mosses (*P. patens*, bryophyte), spike moss (*S. moellendorffii*, lycophyte) and white spruce (*P. glauca*), suggesting that this clade emerged later than the other six clades and presumably has distinct and possibly more specific and specialized functions [23, 46].

The high sequence diversification of the NAC family, especially the C-terminal domain, suggests that the function of this family has also been diversified. This is supported by previous reports on NAC proteins involved in various aspects of plant growth, development and stress responses.

The NAC-encoding genes that are evolutionarily closely related often exert similar functions [46, 80]. In the phylogenetic analysis performed in maritime pine, PpNAC2 and PpNAC3 clustered within the clade NAC-a, suggesting that these TFs may play similar roles to the *Arabidopsis* drought-inducible ATAF1 and ATAF2 [40, 41].

Expression of *PpNAC2* and *PpNAC3* was detected in all tissues tested by qPCR, with higher expression levels of *PpNAC2* in cotyledons and hypocotyls of seedlings, while gene expression levels of *PpNAC3* were particularly abundant in seedling roots (Fig. 3b). This expression pattern suggests that the two genes may perform non-redundant functions in pine seedlings. The closest homologues in *Arabidopsis*, *ATAF1* and *ATAF2*, were also expressed in all tissues; however, according to data derived from Genevestigator, *ATAF1* showed higher expression levels in roots, while *ATAF2* was detected at a higher level in cotyledons and leaves. This transcript distribution pattern in *Arabidopsis* is quite similar to the observed expression pattern of *PpNAC3* and *PpNAC2* in pine. Different reports in the literature have shown that NAC transcription factors play important roles in plant growth, development, hormone signaling and the plant stress response. ATAF1 acts as a negative regulator of ABA signaling but induces JA/ET-associated defense signaling marker genes [31]. However, ATAF2 is induced by dehydration as well as by JA and SA [40]. ANAC19, ANAC055 and RD26 (ANAC072) are induced by drought, high salinity and/or abscisic acid, and the overexpression of these genes up-regulates the expression of several stress-related genes, resulting in the enhancement of plant tolerance to drought stress [38]. Similarly, our study indicates that *PpNAC2* and *PpNAC3* are induced by multiple stress treatments, including salinity, cold and mechanical wounding (Fig. 4). These results suggested that *PpNAC2* and *PpNAC3* may be involved in the general response of *P. pinaster* to abiotic stress. Interestingly, both genes were strongly induced by wounding and MeJA treatments in the seedlings. The role of MeJA as a transportable intercellular molecule that can move from leaves to roots or to other tissues has been established in *Arabidopsis* and in plants of the *Solanaceae* family [81, 82]. This may explain the rapid response of *PpNAC2* and *PpNAC3* genes in hypocotyls following the application of MeJA to the cotyledons. The very rapid accumulation of *PpNAC3* transcripts observed two hours after treatment also suggests that *PpNAC3* is particularly involved in short-term responses to stress*.* The genes *StNAC* in potato, *OsNAC6* in rice, and *ATAF1* and *ATAF2* in *Arabidopsis*, all with high levels of homology to *PpNAC2* and *PpNAC3*, are upregulated by the wounding/pathogenesis-related phytohormone MeJA [40, 83, 84]. Many NAC transcription factors respond to multiple stress signals, and their protein products may participate in the regulation of several apparently unrelated processes as either negative or positive regulators [85].

The analysis of the promoter regions of *PpNAC2* and *PpNAC3* was a pre-requisite to further understand the regulatory control of the stress-inducible expression of these two genes (Fig. 5). Both promoters had several previously characterized stress-responsive *cis*-elements, including DPBF1 (ABA-responsive element) and MYB binding sites [86–88]. Other elements known to be responsive to environmental stimuli, such as light, pathogens, dehydration and low temperature (specifically, W-boxes [89] and GCC-boxes [90]) were also present. W-boxes and GCC-boxes are known as recognition sites for WRKY and ERF transcription factors, respectively. The rapid and transient accumulation of *PpNAC2* and *PpNAC3* indicates their potential as early regulators in the response to wounding/MeJA. The *in silico* analysis of a putative *cis*-element in the *PpNAC2* and *PpNAC3* promoters also showed three E-boxes in *PpNAC2* and six E-boxes in *PpNAC3* (Fig. 5). The E-box elements are commonly found on the promoters of genes that respond to MeJA, and they are well-characterized binding sites for bHLH transcription factors in plants. In general, the genes containing predicted stress-responsive *cis*-elements are actually induced by the corresponding stresses and we were particularly interested in the functional analysis of the transcriptional response of *PpNAC3* to MeJA. The functional analysis of conifer genes has been studied in other model plant species because of the long regeneration times and the technical complexity of producing transgenic trees [91, 92]. In this work, we studied the transcriptional regulation of the *PpNAC3* promoter using *Agrobacterium*-mediated transient expression in *N. benthamiana* leaves. This is a simple and efficient method for the quantitative analysis of plant promoters *in vivo* [61, 62]. Our results suggest that the E-boxes contained in the *PpNAC3* proximal promoter region were transactivated by MeJA treatment in *N. benthamiana* leaves (Fig. 6c). This response disappeared when the *N. benthamiana* plants were silenced for the endogenous *NbbHLH2* gene (Fig. 7c). These results suggest that jasmonic acid induction of the *PpNAC3* promoter is mediated by NbbHLH2 proteins. The *PpNAC3* promoter fragment used in this study appears to contain most elements for proper *PpNAC3* expression, and furthermore, these elements are also recognized in *N. benthamiana* despite the phylogenetic and evolutionary differences between these species.

Notably, although *PpNAC3* is a gene with low expression in pine seedlings, its expression is rapidly and strongly increased in response to MeJA and to several stresses. This raises the possibility of using the *PpNAC3* promoter as a potential MeJA-inducible heterologous promoter, for use as a biotechnological tool to drive gene expression in conifers and other plant species.

## Methods

### Plant material and growth conditions

*Pinus pinaster* Ait. seeds were provided by the Centro de Recursos Genéticos Forestales “El Serranillo” (Ministerio de Medio Ambiente y Medio Rural y Marino, Spain). Seeds were imbibed in distilled water for 24 h under continuous aeration and germinated and grown with vermiculite as a substrate under a growth regime of 16 h light/ 8 h dark at 23 ± 1 °C. Cotyledons, hypocotyls and root samples of pine seedlings were collected separately, frozen in liquid nitrogen and stored at −80 °C until use.

*Nicotiana benthamiana L.* seeds were sown and grown in pots and maintained under a 16 h light/8 h dark photoperiod at 24 °C for 6 weeks.

### Stresses treatments

Four-weeks-old plantlets were used to conduct the stress treatments. Mechanical wounding was conducted by puncturing the hypocotyl with forceps and cutting one-third of the cotyledons. ABA (50 μM) and MeJA (100 μM) solutions were sprayed onto the aerial portion; controls were sprayed only with water [93, 94]. Cotyledons, hypocotyls and roots were harvested for analysis after 0 h, 2 h, 8 h and 24 h of treatment. For NaCl stress, the plantlets were grown in vermiculite and irrigated with a water solution containing 250 mM NaCl; controls were irrigated with water. For the low-temperature (8 °C) treatment, seedlings grown in a chamber at 24 °C were transferred for 48 h at 8 °C, while the control plants were kept at 24 °C. NaCl-treated and cold-treated plants were harvested for analysis at 0 h, 24 h and 48 h after treatment. Three biological replicates for each treatment were collected. Tissue samples were collected separately from the biological replicates, frozen in liquid nitrogen and stored at −80 °C until analysis.

### Cloning of PpNAC2 and PpNAC3

Full-length *PpNAC2* and *PpNAC3* cDNAs were cloned from pine seedling hypocotyl RNAs by reverse transcription-PCR (RT-PCR) using primers designed from *P. pinaster* sequences (SustainpineDB). Pine genomic DNA was isolated using the CTAB method [95] and the promoter sequences of both genes were amplified using the GenomeWalker™ Universal kit (Clontech Laboratories, Mountain View, CA, USA). A 661-bp region of the PpNAC2 promoter and a 1115-bp region of the PpNAC3 promoter were isolated using the Advantage Genomic Polymerase mix (Clontech Laboratories, Mountain View, CA, USA). All the primers are listed in Additional file 5: Table S1. The cDNA and genomic sequences were inserted into the pGEM-3Zf (+) cloning vector and sequenced on both strands using CEQ 8000 Genetic Analysis System (Beckman Coulter, Madrid, Spain).

### Plasmids construction for PpNAC3 Promoter analysis

The pBI121 vector was modified by replacing the GUS reporter gene with a functional GFP gene, thereby placing the GFP gene under the control of the CaMV 35S promoter (p35S-GFP). A 1,165 bp genomic fragment upstream of the ATG (+1) of the PpNAC3 gene was cloned into the pGEM-3Zf (+). It was amplified using PCR with specific flanking primers containing restriction sites (Additional file 4: Table S1). After digestion, the product was inserted upstream the GFP gene in the binary vector p35S-GFP replacing the 35S promoter (P_NAC3_-GFP). The recombinant plasmid was introduced into *Agrobacterium tumefaciens* C58C1 and the overnight cell cultures were centrifuged and resuspended in infiltration media containing 10 mM MES-KOH (pH5.6) and 0.15 mM acetosyringone. The optical density at 600 nm was adjusted to 0.5 before infiltration of the leaves of 4-5-week-old *N. benthamiana* plants. The p35S-GPF plasmid was used as a positive control, and plasmid without the 35S promoter (P0-GFP) or MES were used as the negative controls. At 48 h after infiltration, *N. benthamiana* leaves were treated with 100 μM MeJA solution or with water as a control. Three leaf discs (10 mm) of three independents plants were used for each time point tested. The experiments were repeated at least three times.

### Virus Induced Gene Silencing (VIGS) experiments

A virus induced gene silencing (VIGS) system was used to transiently silence the transcription factor MYC2. We amplified a 400-bp fragment of the *N. benthamiana* bHLH2 (ADH04263.1) gene using specific Gateway adapter primers (Additional file 4: Table S1) recombined into pDONR207 (Invitrogen) and cloned into the VIGS Gateway-adapted destination vector pTRVGW. Constructs were verified by sequencing using a CEQ 8000 (Beckman Coulter, Spain). Subsequently, we transformed the *A. tumefaciens* strain C58C1 with the empty plasmid (pTRV-control) or with a plasmid harbouring the insert (pTRV-NbbHLH2) and incubated the bacteria at 28 °C for two days. Silencing in *N. benthamiana* leaves was performed as described by [66]. Fifteen-day-old seedlings were infiltrated with cultures containing pTRV-control or pTRV-*NbbHLH2* prepared in a 1:1 mix of each with the helper plasmid pTRV-1, both with an optical density at 600 nm of 0.1. The plants were kept in the growth chamber under 16 h light/ 8 h dark regime at 24 °C. We monitored the spread of silencing using control plants infiltrated with the pTRV-*PDS* construct which induced leaf bleaching, while the efficiency of silencing of the corresponding gene was determined by measuring transcript abundance by qPCR using specific primers (Additional file 4: Table S1). Four weeks after silencing, *N. benthamiana* plant leaves were agroinfiltrated with P_NAC3_-GFP, P35S-GFP and P0-GFP constructs; after 48 h the leaves were treated either with 100 μM MeJA solution or with water as a control. Three leaf discs (10 mm) of three independent plants were used for each time point tested. The experiments were repeated in triplicate, and results from a representative experiment are shown.

### RNA isolation and qPCR

Total RNA was extracted following the protocol described previously [96]. RNA samples were treated with RQ1 RNase-Free DNase (Promega), and cDNA synthesis was performed with iScript Reverse Transcription Supermix (Bio-Rad) in the presence of oligo (dT) or random primers. Real-time PCR was performed in a reaction volume of 10 μl containing cDNA (10 ng) and 5 μl of SsoFast EvaGreen Supermix (Bio-Rad) using a CFX384 Real- Time System C1000 Thermal Cycler (Bio-Rad) with annealing at 60 °C for 10 s and extension at 72 °C for 15 s. The reactions were run for 40 cycles, and after the final cycle, a melting curve was performed to verify the reaction specificity. Actin, elongation factor-1-alpha (EF1-α), and 40S ribosomal protein were used as reference genes in pine, and actin was used as a reference gene in *N. benthamiana* samples. The fluorescence of the PCR products was continuously monitored using an iQ5 cycler (Bio-Rad), and relative gene expression was estimated as previously described [94]. The gene-specific primers used are described in Additional file 4: Table S1.

### Bioinformatics

#### Database search and phylogenetic analysis

The program Clustal X, version 1.83, was used for multiple sequence alignments of the full-length protein sequences, including the highly conserved N-terminal NAC domain and the more divergent C-terminal domain. The unrooted phylogenetic trees were constructed with MEGA 4.0 using the Neighbor-Joining (NJ) method, and the bootstrap test was carried out with 1000 iterations. A pairwise gap deletion mode was used to ensure that the more divergent C-terminal domains could contribute to the topology of the NJ tree.

### Motif identification

Protein sequence motifs were identified using the MEME (Multiple Expectation Maximization for Motif Elicitation) program version 4.9.1 (http://meme.nbcr.net/meme/cgi-bin/meme.cgi, [45]), with the following parameters: number of repetitions, any; maximum number of motifs, 50; and optimum width of the motif between 6 and 50 residues. The motif profile for each protein is presented schematically.

### Identification of *cis*-regulatory elements

For the *in silico* study of PpNAC2 and PpNAC3 promoter sequences and to determine the *cis*-acting regulatory elements a search using the plant databases PlantCARE (http://bioinformatics.psb.ugent.be/webtools/plantcare/html/) and PLACE (http://www.dna.affrc.go.jp/PLACE/) was conducted.

## Conclusions

In summary, we have studied the composition of the NAC transcription factors family in maritime pine and classified 37 NAC genes into six subfamilies. The largest group included 12 genes stress-related. We have characterized the expression profiles of two genes of this group: *PpNAC2* and *PpNAC3*, in response to various types of stress. Both genes were strongly induced by methyl jasmonate, mechanical wounding and high salinity. In addition, the study *in silico* of both promoters revealed the existence of cis-elements such as E-boxes, which are commonly found in promoters that respond to jasmonate and are binding sites for bHLH proteins. Using a transient expression assay in *N. benthamiana* leaves we found that the promoter of *PpNAC3* was rapidly induced upon MeJA treatment however, this response disappeared in plants in which the internal transcription factor NbbHLH2 had been silenced. Our results suggest that *PpNAC2* and *PpNAC3* encode stress-responsive NAC transcription factors involved in the jasmonate response in maritime pine. Furthermore, our results support the idea that NbbHLH2 proteins can mediate jasmonic acid induction of *PpNAC3* promoter suggesting that the jasmonate signaling pathway could be conserved between angiosperms and gymnosperms. These findings raise the possibility of using *PpNAC3* promoter as a biotechnological tool to drive MeJA-inducible expression in conifers and other plants.

## Additional files

Additional file 1:
**Names, gene accession numbers and sequences of the** ***P. pinaster*** **NAC proteins.** (XLS 37 kb) Additional file 2:
**Amino acid sequence logos of 21 motifs identified in PpNAC proteins using MEME.** Data shown are the number of proteins containing each motif, the width, the E-value, the domain in which it is located, and the annotation of each motif is indicated. (XLS 379 kb) Additional file 3: Figure S1. Sequence alignment of PpNAC proteins from *P. pinaster*. Amino acid motifs (A-E) in the NAC proteins are represented by colored boxes. Secondary structure elements (β, α, helices) of the NAC domain are indicated above the alignment. (TIFF 544 kb) Additional file 4: Table S2. Gene accession numbers, names and references for the genes used in the phylogenetic analysis. Phylogenetic groups according to Shen et al. (2009) [46] and the groups generated in this study are indicated. (DOC 95 kb) Additional file 5: Table S1. Oligonucleotides used in this work. (DOC 50 kb)
