# Supplementary material for: The NAC transcription factor family in maritime pine (Pinus Pinaster): molecular regulation of two genes involved in stress responses
Source: BMC Plant Biol. 2015 Oct 24;15:254. doi: 10.1186/s12870-015-0640-0 (PMC4619436; doi:10.1186/s12870-015-0640-0)
Supplement: Additional file 4: Table S2. — Gene accession numbers, names and references for the genes used in the phylogenetic analysis. Phylogenetic groups according to Shen et al. (2009) [46] and the groups generated in this study are indicated. (DOC 95 kb) [file 12870_2015_640_MOESM4_ESM.doc]

**Additional file 4: Table S2**. Gene accession numbers, names and references for the genes used in the phylogenetic analysis. Phylogenetic groups according to Shen et al. (2009) and the groups generated in this study are indicated.

| Species | Accession no. | Name | Phylogenetic group | **Reference** |
| --- | --- | --- | --- | --- |
| Arabidopsis thaliana |  |  |  |  |
|  | AT3G15510.1 | AtNAC2 | A | **He et al. 2005** |
|  | AT1G01720.1 | ATAF1 | A | **Lu et al., 2007** |
|  | AT5G08790.1 | ATAF2 | A | **Delessert et al. 2005** |
|  | AT1G77450.1 | ANAC032 | A |  |
|  | AT5G04410.1 | ANAC078 | B | **Nishizawa et al. 2006** |
|  | AT1G62700.1 | VND5 | C | **Kubo et al. 2005** |
|  | AT4G36160.1 | VND2 | C | **Kubo et al. 2005** |
|  | AT2G18060.1 | VND1 | C | **Kubo et al. 2005** |
|  | AT1G79580.1 | SMB | C | **Willemsen et al. 2008** |
|  | AT3G61910.1 | NST2 | C | **Mitsuda et al. 2005** |
|  | AT2G46770.1 | NST1 | C | **Mitsuda et al. 2005** |
|  | AT1G32770.1 | SND1 | C | **Zhong et al. 2006** |
|  | AT5G39610.1 | ORE | D | **He et al. 2005; Kim et al. 2009** |
|  | AT3G15170.1 | CUC1 | D | **Takada et al. 2001** |
|  | AT5G53950.1 | CUC2 | D | **Aida et al. 1997** |
|  | AT1G76420.1 | CUC3 | D | **Vroemen et al. 2003** |
|  | AT1G56010.2 | NAC1 | D | **Xie et al. 2000.** |
|  | AT2G43000.1 | ANAC042 | E |  |
|  | AT1G26870.1 | FEZ | E | **Willemsen et al. 2008** |
|  | AT2G02450.2 | ANAC34/ANAC035 | E |  |
|  | AT5G41090.1 | ANAC095 | F |  |
|  | AT5G66300.1 | ANAC105 | F |  |
|  | AT4G28500.1 | SND2 | G | **Zhong et al. 2008** |
|  | AT1G28470.1 | SND3 | G | **Zhong et al. 2008** |
|  | AT1G64105.1 | ANAC027 | H |  |
|  | AT1G03490.1 | ANAC006 | H |  |
| Physcomitrella patens |  |  |  |  |
|  | A9T4L6 | PpaNAC09 | A |  |
|  | A9SFD1 | PpaNAC35 | B |  |
|  | A9TC00 | PpaNAC13 | C |  |
|  | A9T3H2 | PpaNAC07 | D |  |
|  | A9TKG7 | PpaNAC30 | G |  |
| Picea abies |  |  |  |  |
|  | HM68414 | PaNAC01 | D | **Larsson et al. 2012** |
|  | HM638415 | PaNAC02 | D | **Larsson et al. 2012** |
| Picea glauca |  |  |  |  |
|  | BT115957.1 | PgNAC04 | A |  |
|  | BT115770.1 | PgNAC07 | A |  |
|  | BT116552.1 | PgNAC08 | B |  |
|  | BT103829.1 | PgNAC12 | B |  |
|  | BT102049.1 | PgNAC14 | C |  |
|  | BT101915.1 | PgNAC15 | C |  |
|  | **BT109581.1** | **PgNAC16** | **E** |  |
