# Supplementary material for: The NAC transcription factor family in maritime pine (Pinus Pinaster): molecular regulation of two genes involved in stress responses
Source: BMC Plant Biol. 2015 Oct 24;15:254. doi: 10.1186/s12870-015-0640-0 (PMC4619436; doi:10.1186/s12870-015-0640-0)
Supplement: Additional file 5: Table S1. — Oligonucleotides used in this work. (DOC 50 kb) [file 12870_2015_640_MOESM5_ESM.doc]

**Additional file 5: Table S1**. Oligonucleotides used in this work.

| Name | Sequence |
| --- | --- |
| Fw1 PpNAC2 | 5´- GCTAGTTCTGAAGGAATTGGC-3´ |
| Fw qPCR PpNAC2 | 5´- GGGGATTGAAAGTCGAGTCTA- 3´ |
| Rev qPCR PpNAC2 | 5´- TGAGGAGGCTTGCACATACA- 3´ |
| Fw1 PpNAC3 | 5´- ATGGGTATGAGTAGAAGAAATG- 3´ |
| Fw qPCR PpNAC3 | 5´- CCACTTCAGACTACCTACCAAG- 3´ |
| Rev qPCR PpNAC3 | 5´- GAAGCTAAAACGAGAGATTCAG-3´ |
| Pm1 PpNAC2 | 5´- GGCAGTGAGGCAGCCTTCTTGCACAAG- 3´ |
| Pm2 PpNAC2 | 5´- GGTAAATTCAGCTGGGCCTCTGCATCC- 3´ |
| Pm3 PpNAC3 | 5´- CTTGCACAAGTAATGCACTACCAGCTCG- 3´ |
| Fw1 Pm *(PstI)* | 5´- CG*CTGCAG*GACAATATGGAAT- 3´ |
| Fw2 Pm *(PstI)* | 5´- CG*CTGCAG*CAGTTCCATACCA- 3´ |
| Fw3 Pm *(PstI)* | 5´- CG*CTGCAG*AGAGCATAATTCC |
| Fw4 Pm *(PstI)* | 5´- CG*CTGCAG*ACATGAATATTGG |
| Rev Pm *(BamHI)* | 5´- CG*GGATCC*GTTTATCCTAGTG |
| Fw GFP *(BamHI)* | 5´- CA*GGATCC*ATG GTGAGCAAGGGCG- 3´ |
| Rev GFP *(SacI)* | 5´- CC*GAGCTC*TTACTTGTACAGCTCG - 3´ |
| Fw qPCR GFP | 5´- CCACATGAAGCAGCACGAC- 3´ |
| Rev qPCR GFP | 5´- TGTCGCCCTCGAACTTCAC- 3´ |
| Fw qPCR EF1α | 5´- TGCTGTTGGAGTCATCAAGG-3´ |
| Rev qPCR EF1α | 5´- CTCGTGCATCAGAATCAGACA-3´ |
| Fw qPCR 40S | 5´- TCTTGAGAGTGGAGAATGGG- 3´ |
| Rev qPCR 40S | 5´- CGCATCAGTCATACTCACCT- 3´ |
| Fw qPCR *NbActin* | 5´- AGCCACACAGTTCCCATCTAT- 3´ |
| Rev qPCR *NbActin* | 5´- TAGGGATGTGAAGGAGAAGTT- 3´ |
| Fw qPCR *NbPR4* | 5´- GGCCAAGATTCCTGTGGTAGAT- 3´ |
| Rev qPCR *NbPR4* | 5´- CACTGTTGTTTGAGTTCCTGTTC -3´ |
| NbMYC attB1 | 5´- AAAAAGCAGGCTTAAGCAATACAACATCCGACG- 3´ |
| NbMYC attB2 | 5´- AGAAAGCTGGGTCTCAGCTCCCGGAGAACTTT- 3´ |
| attB1 | 5´- ACAAGTTTGTACAAAAAGCAGGCT- 3´ |
| attB2 | 5´- ACCACTTTGTACAAGAAAGCTGGGT- 3´ |
| Fw qPCR NbMYC | 5´- GCCAACAAGGGATCAAACTATACCG- 3´ |
| Rev qPCR NbMYC | 5´- AGTCCAATTCCATGAGAGCGGC- 3´ |
